# An examination of energy efficiency retrofit scheme applications by low-income households in Ireland^☆^^☆☆^

**DOI:** 10.1016/j.heliyon.2021.e08205

**Published:** 2021-10-18

**Authors:** Arya Pillai, Miguel Tovar Reaños, John Curtis

**Affiliations:** a Economic and Social Research Institute, Sir John Rogerson's Quay, Dublin, Ireland; b Trinity College Dublin, Dublin, Ireland

**Keywords:** Energy efficiency, Energy retrofits, Retrofit intensity, Vulnerable households, Abandonments

## Abstract

This paper studies the determinants of why low-income households in Ireland abandon energy efficiency retrofit applications using administrative data from a targeted energy efficiency grant. By applying for the scheme, the applicants overcome any financial barriers for undertaking retrofits and demonstrate their willingness to improve the energy efficiency of their dwellings. Hence this study contributes to the scarce literature on non-financial barriers preventing low-income households from undertaking energy efficiency retrofits. Contrary to previous findings, we find that the higher the number of retrofits to be implemented, the lower the probability of households abandoning their applications. We also find that planning to undertake retrofits such as ventilation, which can significantly improve the health and safety standards of the dwelling, is associated with a higher probability of abandonment. Both findings indicate the presence of key behavioural and informational barriers which prevent low-income households from fully comprehending the purpose or benefits of proposed energy efficient retrofits. Our findings also suggest that higher grant expenditure on dwellings with poor pre-works energy efficiency rating and on retrofits such as attic insulation and heating system upgrades may have the highest energy efficiency improvements per unit of expenditure. Within the constraints of limited budgets for retrofit grant supports, this research can inform the redesign of grant schemes to achieve the greatest aggregate improvements in residential building energy efficiency.

## 1 Introduction

Apart from contributing significantly to the campaign against global warming, reducing energy inefficiency in the dwellings of low-income households can help break the cycle of poverty perpetuated by higher energy costs. Previous studies show that poorer households often forgo some life necessities such as food and medicines to cover high energy bills (Anderson et al., 2010; Raissi and Reames, 2020; Healy and Clinch, 2004). Buildings' energy efficiency can also affect physical and mental health and the well-being of residents (Campbell et al., 2014; European Parliament, 2016). Low adoption of energy efficiency retrofits by low-income households is attributed to four barriers: economic, informational, behavioural and, administrative (Sorrell et al., 2004, Sorrell et al., 2000; Raissi and Reames, 2020; European Parliament, 2016). The economic barriers include lack of savings and inability to obtain credit to upgrade inefficient appliances and infrastructure. Poorer households, in the face of uncertain future earnings, are also highly risk averse (Ozaki, 2011; Chen et al., 2017). Across many European countries, subsidies for the adoption of energy saving technologies are core policy instruments to protect vulnerable households and overcome financial barriers for the adoption of these technologies (e.g. Kyprianou et al., 2019; Drivas et al., 2019). However, there are households who make a decision to engage in an energy efficiency retrofit free from financial constraints (i.e., have fully funded grant support) but abandon their retrofit applications due to non-financial barriers, which are often harder to identify. While the abandonment of energy efficiency scheme applications has already been investigated (e.g. Collins and Curtis, 2017a), the abandonment of programs targeting vulnerable households has received little attention in the existing literature.

This study utilises an administrative dataset comprising application and processing information related to a targeted energy efficiency grant in Ireland. By applying for this scheme, low-income households overcome the financial barriers cited earlier. However, the abandonment of retrofit applications shows that significant barriers exist for low-income households even in the absence of financial barriers. The main objective of this study is to better understand the factors associated with the abandonment of retrofit grant applications in the absence of financial barriers. Additionally, we quantify how improvements in building energy efficiency among low-income households are associated with retrofit measure type and building attributes. This paper adds to the literature on adoption of energy efficient retrofits by low-income households.

Aside from financial constraints to the adoption of energy saving technologies, several other barriers may prevent low-income households from undertaking retrofits. Behavioural and informational barriers may arise due to the lower educational status of many low-income households (Abrahamse and Steg, 2009, Abrahamse and Steg, 2011; Achtnicht and Madlener, 2014; Fylan et al., 2016; Chen et al., 2017). Barriers can similarly be higher for low-income households considering their precarious employment conditions. Poorer households are often less aware of their energy consumption metrics and the potential savings which can be obtained from upgrading (Chen et al., 2017; Day and Gunderson, 2015). Many low-income households further fail to differentiate between maintenance costs and energy efficiency improvements (Chen et al., 2017; Poortinga et al., 2004). Lack of awareness about the support schemes available can be another important barrier in undertaking retrofits. In some cases, low-income households do not participate because a particular scheme may not be addressing their specific vulnerability or they misunderstand scheme eligibility conditions (Raissi and Reames, 2020). Households may also forgo lengthy application procedures which are often accompanied by disruptive practical arrangements needed for retrofits due to short-term practicalities (Raissi and Reames, 2020; Brown et al., 2019). Such hidden costs raise significant barriers to the adoption of energy efficiency measures. These barriers can be more significant in the adoption of retrofit measures which cause more disruption (Collins and Curtis, 2017a).

Studies conducted on low and higher income households identify household and dwelling level factors that influences adoption of retrofits and participation in schemes to improve energy efficiency (Achtnicht and Madlener, 2014; Morton et al., 2018; Trotta, 2018). When it comes to low-income households, older homeowners have a higher likelihood of adopting energy retrofits across the EU (Schleich, 2019). This finding is opposite to the findings by Achtnicht and Madlener (2014) and Morton et al. (2018) from their studies including higher income households. Low-income households who are concerned with their energy cost and those who are more environmentally conscious have a higher likelihood of adopting energy efficient retrofits (Schleich, 2019). Detached homes and older dwellings have a higher likelihood of having undertaken retrofits among low-income households (Schleich, 2019) similar to higher income households (Achtnicht and Madlener, 2014; Trotta, 2018; Morton et al., 2018). However, Trotta (2018) notes that the relationship between age of the dwelling and likelihood of undertaking retrofits is not clear. Wolske (2020) argues that low, medium and high-income adopters of energy efficient retrofits, such as solar panels, are more similar than not and often fit the profile of early adopters. The relationship between likelihood of undertaking retrofits and education is weaker for higher income households when controlled for knowledge of climate change (Achtnicht and Madlener, 2014). Morton et al. (2018) in their study on retrofit grants targeting households in UK found that smaller households and households with self-employed members are less likely to undertake retrofits. Couples with children are more likely to invest in energy efficient retrofits in higher income households compared to single parents (Trotta, 2018). While previous literature on low-income households identifies different socioeconomic drivers of adoption of energy efficient retrofits, research on the abandonment of applications from programmes targeting low-income households has not received enough attention.

Outcome evaluations of retrofit grants can be important in decision-making regarding the continuation of funding for such schemes or search for alternate strategies for energy efficiency improvements (Fylan et al., 2016). Previous research finds that energy efficiency retrofit grants can significantly improve energy efficiency in private dwellings (Hoicka et al., 2014; Webber et al., 2015; Collins and Curtis, 2017b). Rau et al. (2020) find that government or community assisted energy retrofits in Ireland bring better thermal comfort and reduction of energy use. However, they argue that this must be coupled with changes in energy cultures to obtain best value for investment made. Energy efficiency grants are especially important in case of homeowners falling into the lower income quartiles (Schleich, 2019; Wilson et al., 2019). Literature is sparse on how grants specifically targeting low-income households improve energy efficiency. When it comes to the quantification of the savings attributed to programs that offer grants for the adoption of energy efficiency technologies to vulnerable households, Beagon et al. (2018) and Hernández and Phillips (2015), utilising data from Ireland and USA, find that participation in energy efficiency programs reduce energy consumption. Similar to the findings of Rau et al. (2020), studies conducted on social housing retrofit assistance and grants for low-income households in UK find that home conditions were significantly improved, and energy use was reduced by the programmes (Liddell, 2015; Elsharkawy and Rutherford, 2018).

The inability of households to afford a warm home is a growing concern in many countries. In Europe, Galvin (2019) finds that income inequality is an important driver of the proportion of people in fuel poverty, while Halkos and Gkampoura (2021) find that declining GDP will aggravate fuel poverty. Improvements in energy efficiency can reduce the proportion of households living in fuel poverty (see Tovar Reaños, 2021).

In this study we utilise the administrative dataset of a residential energy efficiency grant scheme targeting low-income households where the full cost of the retrofit is paid by the grant. The analysis contributes to a relatively sparse literature on energy efficiency retrofits among low-income households. Additionally, given the 100% grant funding the analysis can set aside budgetary constraints and investigate non-financial barriers facing low-income households from undertaking energy efficiency retrofit. Furthermore, we quantify improvements in energy efficiency metrics and identify retrofit types (i.e., insulation, ventilation etc) associated with the greater efficiency gains. Insights from this study will help improve the design and marketing of retrofit schemes to improve the uptake of retrofits by low-income households.

## 2 Data and methodology

### 2.1 Data

**Figure 1:**
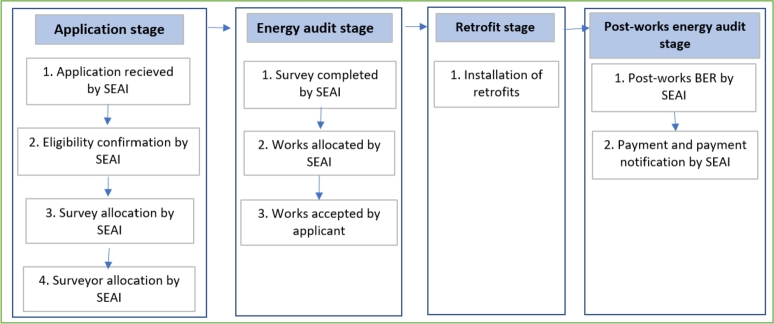
Scheme process as a flow chart.

This study utilises administrative data consisting of the application and processing information related to the Better Energy Warmer Homes Scheme (BEWHS), which provides free energy efficiency upgrades to low-income households in Ireland. Scheme eligibility is confined to owner-occupier households that are recipients of at least one of six social welfare schemes targeting low-income households. Only dwellings built before 2006 can apply for the scheme. The scheme administrator, Sustainable Energy Authority of Ireland (SEAI), rather than the household applicant, decides the type of energy efficiency retrofit measures to be installed for each dwelling following a building energy audit. The scheme follows 4 main steps: application, energy survey, retrofit works, and post-works energy audit stages, as illustrated by the flowchart in Fig. 1. Once the applicant files an application, the SEAI assess the eligibility of the applicant and allocate the property for survey. An SEAI surveyor then decides what works needs to be implemented in the residence, and the list of proposed retrofits is notified to the applicant for their acceptance. The applicant cannot choose a subset of the notified retrofit works, but they can withdraw from the scheme if they are not willing to undertake the entirety of the notified works. Once approved by the applicants the SEAI assigns the installation to their approved contractor. Once the installation is complete a post-work energy rating is conducted, and payment is made by the SEAI directly to the contractor. The applicants can apply for the scheme again if their previous application was not approved or withdrawn, however they can receive the grant only once.

There are 35,395 unique applications (or dwellings) in the dataset used for this study. The raw data had 49,564 observations. This included cancelled applications (10,426) and entries with missing information (2,845). The cancelled applications are applications which are not eligible for the scheme and hence not relevant for this study and are therefore excluded. Also excluded are 898 applications made in the year 2020, as the natural conclusion of those applications was not available at time of data access. As the analysis is based on an anonymised administrative dataset the study did not require ethical approval. The dataset includes applications at various stages of processing, for example, those awaiting eligibility letters (early application stage) to those whose grant payments are processed (advanced post-works stage). An application is considered ‘abandoned’ when its status is listed as cancelled on the instruction of the homeowner or as those who cannot be contacted 30 days after a notice was issued by SEAI. There are 3,312 ‘abandoned’ applications in the dataset (9%). Descriptive statistics of these variables are provided in Table 1 and a county-wise breakdown of applications and retrofits undertaken is given in Table 7. The average number of days taken to complete each stage is given in Table 2.

**Table 1:** Descriptive statistics.

Variable	Frequency	Proportion
**Total applications**	**35,395**	**100.00**
Application status		
Processing	5,893	0.17
Abandoned	3,312	0.09
Works completed	26,190	0.74
Person employed	9,540	0.27
Dwelling type		
Apartment	232	0.01
House	31,054	0.88
Others	4,109	0.11
Year of construction		
Before 1920s	2,671	0.07
1920s-1930s	2,677	0.08
1940s-1960s	5,939	0.17
1960s-1980s	14,165	0.4
1990s - 2006	9,943	0.28
Season		
Summer	7,142	0.2
Autumn	8,767	0.25
Winter	7,932	0.22
Spring	11,554	0.33
Retrofit type dummy		
Heating system	3,145	0.09
Wall insulation	20,809	0.59
Attic insulation	22,971	0.65
Lighting	26,440	0.75
Network costs	1,508	0.04
Ventilation	29,170	0.82
Miscellaneous	18,778	0.53

**Table 2:** Duration of each stage of processing.

Stage	Mean (days)	Standard deviation (days)
Application stage	128	194
Energy audit stage	62.1	284
Retrofit stage	38.2	419
Post-works energy audit stage	37.7	132

The retrofit type categories in the data include heating system upgrades, attic insulation, wall insulation, network costs, ventilation, lighting and miscellaneous (see Table 4 for a detailed list of retrofit measures undertaken). The miscellaneous category includes retrofits related to draught proofing and hot water cylinder jacket installation. An indicator for seasonality is constructed to study the effect of the timing of an application on its advancement. Letter grades for Building Energy Rating (BER) ratings, from A (high energy efficiency) to G (low energy efficiency) is available for all properties pre-retrofit. The change in BER between pre and post energy efficiency retrofit denominated in kWh/m^2^/year is available for only a subset of applications (2,447 observations). A property's BER is undertaken by SEAI's registered BER assessors (SEAI, 2020). Summary statistics of these variables and other variables related to household and dwelling sizes are provided in Table 3.

**Table 3:** Summary statistics for indicators used in the study.

Indicators	Mean	SD
Household size (count)	2.27	1.47
Duration of processing (months)	0.13	0.47
Dwelling size (sqm)	115.14	49.18
Retrofits per dwelling (count)	8.58	3
Change in BER (kWh/m^2^/year)	68.59	133.29
Grant amount (euros)	4070.26	5761.83
Grant amount by type of retrofit		
Heating system	357.85	1793.81
Wall insulation	1442.48	3959.97
Attic insulation	520.75	609.56
Lighting	14.27	10.82
Network costs	0.25	9.66
Ventilation	690.36	673.69
Miscellaneous	16.13	26.11

**Table 4:** List of retrofit activities undertaken in each broad retrofit category.

Heating system	Motorised valve and wiring
	Boiler servicing
	Hot water cylinder with anode
	Electrical bonding and earthing
	Room/ cylinder stat and wiring
	Separation of zones, hot water and space heating
	Seven day programmer and wiring
	Wireless electronic fuel gauge
	High efficiency gas/ oil boiler
	Oil tank and supply line
	Additional space heating zone
	Cooker hood
	DCV: habitable/ wet room
	Remove boiler, hot water cylinder, oil tank, radiators or solid fuel cooker
	Remove all electric heating and related wiring
	Dual fuel interlinking
	Installation of fire barriers for oil tanks
	Power cleanse of heating system
	Multi-fuel stove with boiler
	MVHR: Habitable or wet room
	Pipe work and valves for boilers
	Radiators
	Removal or replacement of fireplace, flues and tanks
	TRV
Ventilation	Additional roof ventilation
	Air tightness testing
	Wall vent
	Magnetic filtration device
	Mechanical extract vent

Insulation	Draught proofing
	Hot water cylinder jacket

Attic insulation	Attic insulation and top up (100-300 mm)
	Attic storage (5 m^2^)
	Attic hatch

Wall insulation	Cavity insulation bonded bead
	External wall insulation (various types)
	Internal wall insulation
	Loose fibre extraction
	Glazed, sliding or solid room
	Window

Network costs	Network charge for gas connection
	Electrical bonding and earthing
	ESB alteration
	GNI new meter or meter alteration

Lighting	CFL bulbs

Energy advice	Energy advice

### 2.2 Methods

This study employs two separate analytical methods to study the two research questions. These are explained in the next subsections.

#### 2.2.1 Method used for the analysis of abandonment of applications

The first objective of this paper is the determinants of why low-income households in Ireland abandon energy efficiency retrofit applications. We model the probability of abandonment as a function of dwelling, household, and application processing related characteristics. The dependent variable in this case is a dummy variable indicating whether the application was abandoned by the applicant or not. The estimation method employed in this case is a logit regression. We follow the methodology employed by (Greene, 2003).

(1) $$Pr(Abandonment=1|Z_{i})=f(\beta_{0}+\beta_{1}Z_{i}+u_{i})$$

$Z_{i}$ in this case refers to the matrix of household, dwelling, and application related characteristics. Household related characteristics include household size and person employed dummy. Dwelling related characteristics include the year of construction, dwelling type, and dwelling location. Application related characteristics include the total count of planned retrofit measures, duration of processing, year of application and dummy variables for retrofit type. $\beta_{0}$ and $\beta_{1}$ are the estimated parameters and $u_{i}$ is the random error component. *f* is the logistic function. Since logit parameter estimates are difficult to interpret, odds ratios ($OR=e^{\beta }$) are calculated, and the statistical significance is tested as different from OR=1.

#### 2.2.2 Method used for the analysis of post-works efficiency gain

Improvement in energy efficiency is modelled as a function of retrofit measures undertaken and dwelling characteristics. The dependent variable in this case is the change in energy rating (in kWh/$m^{2}$/year), calculated as the difference between post-works and pre-works BER. A large positive value for the dependent variable indicates significant energy efficiency improvement.

(2) $$\Delta BER\;=\beta_{0}+\beta_{1}Z_{i}+u_{i}$$

$Z_{i}$ in this case relates to dwelling related characteristics and retrofit details. Dwelling related characteristics included are dwelling size, year of construction and dwelling type, while retrofit details include expenditure on each retrofit measure type.

## 3 Results and discussion

### 3.1 Factors affecting abandonment of applications

**Figure 2:**
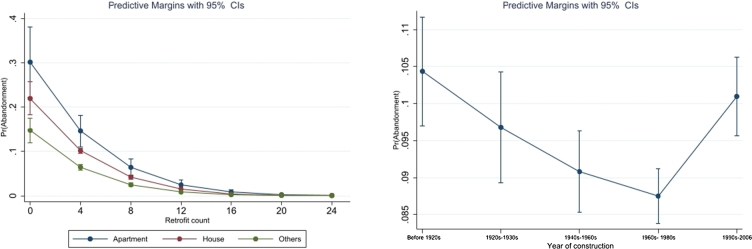
Predicted probabilities of abandonment at various levels of count of retrofits planned and year of construction.

As mentioned in the data section, 9% of applications are abandoned. The dependent variable in this case is a dummy variable indicating whether an application was abandoned or not. The results of regression analysis studying the factors affecting abandonment are presented in Table 5. Two versions of the model are estimated, with and without year and location controls. The justification behind this is to account for changes in the administration of the scheme over time.^1^ The common coefficients are consistent across both models. Model 2 with year and location controls is the preferred model for discussion since it has a lower Akaike information criterion (AIC), which is a measure of the goodness of fit of the model (Wooldridge, 2010). In addition to the odds ratios reported in Table 5, predicted probability estimates are reported in Table 6 to facilitate further discussion. Fig. 2 plots some of the estimated predicted probabilities.

**Table 5:** Factors influencing likelihood of abandonment of applications.

	Dependent variable: abandonment dummy	
	1	2
Retrofit count	0.721***	0.732***
	(0.0187)	(0.0190)
Duration of processing (months)	0.448***	
	(0.0284)	
Dwelling type (ref cat: Houses)		
Apartment	1.820***	1.974***
	(0.373)	(0.431)
Others	0.499***	0.525***
	(0.0354)	(0.0390)
Household size	0.846***	0.850***
	(0.0193)	(0.0202)
Person employed (ref cat: No)	0.614***	0.615***
	(0.0422)	(0.0441)
Season (ref cat: Summer)		
Autumn	0.876*	0.921
	(0.0609)	(0.0676)
Winter	1.119	1.299***
	(0.0834)	(0.102)
Spring	1.462***	1.703***
	(0.0896)	(0.112)
Year built (ref cat: 1990s-2006)		
Before 1920s	1.088	1.069
	(0.0937)	(0.0982)
1920s-1930s	0.893	0.920
	(0.0806)	(0.0888)
1940s-1960s	0.704***	0.813**
	(0.0535)	(0.0668)
1960s-1980s	0.714***	0.759***
	(0.0456)	(0.0520)
Retrofit type dummies		
Heating system	1.090	1.077
	(0.151)	(0.154)
Wall insulation	0.715**	0.658***
	(0.0935)	(0.0868)
Attic insulation	2.291***	2.108***
	(0.263)	(0.245)
Ventilation	5.023***	4.142***
	(0.646)	(0.542)
Network costs	0.221***	0.247***
	(0.0396)	(0.0451)
Lighting	0.0126***	0.0103***
	(0.00133)	(0.00112)
Miscellaneous	1.763***	1.686***
	(0.147)	(0.142)
Duration*Year interaction		
2015		1.654***
		(0.169)
2016		2.025***
		(0.352)
2017		3.704***
		(0.515)
2018		1.050
		(0.111)
2019		0.0169***
		(0.00411)
Constant	1.513***	2.324***
	(0.134)	(0.507)

Year-duration interaction	No	Yes
County dummies	No	Yes
Observations	35,395	35,394
AIC	12460.25	11354.87

Standard error in parentheses; Odds ratios given in the table. *** p<0.01, ** p<0.05, * p<0.1.

**Table 6:** Predicted probabilities of abandonment at various levels of independent variables.

	Predicted probability	Standard error
Retrofit count = 5	0.081***	0.001
Retrofit count = 1	0.166***	0.011
Apartments (ref: House)	0.036**	0.010
Household size (at 6)	0.066***	0.003
Household size (at 1)	0.103 ***	0.002
Person employed (ref: unemployed)	-0.023***	0.003
Season of application (ref: summer)		
Autumn	-0.004	0.003
Winter	0.013***	0.004
Spring	0.026***	0.003
Construction period (ref: pre 1920s)		
1920s-1930s	-0.007	0.005
1940s-1960s	-0.013**	0.010
1960s-1980s	-0.016***	0.004
1990-2006	-0.003	0.004
Retrofit measures (ref: specified measure not installed)		
Heating system upgrade	0.003	0.007
Wall insulation	-0.020***	0.001
Attic insulation	0.036***	0.005
Ventilation retrofit	0.06***	0.005
Networks costs	-0.056***	0.005
Lighting	-0.361 ***	0.012
Miscellaneous	0.025***	0.004

*** p<0.01, ** p<0.05, * p<0.1.

**Table 7:** Distribution of grant application count by type of retrofits undertaken across counties in Ireland.

County	Total applications	Attic insulation	Insulation	Wall insulation	Heating system	Ventilation	Lighting	Network costs	Others
Co. Dublin	7585	5057	3683	3175	961	5905	4827	828	1622
Co. Cork	2979	1875	1402	1723	297	2353	2123	128	608
Co. Donegal	1893	1200	972	1283	163	1610	1510	12	277
Co. Galway	1846	1075	891	1287	129	1554	1442	36	283
Co. Wexford	1842	1093	956	1250	160	1514	1412	44	313
Co. Mayo	1745	1071	934	1290	113	1521	1382	21	221
Co. Meath	1541	1134	930	859	106	1344	1261	52	180
Co. Kildare	1515	1038	883	792	154	1274	1207	27	223
Co. Louth	1256	891	811	842	51	1095	1049	26	143
Co. Wicklow	1189	744	638	648	91	963	897	48	208
Co. Tipperary	1096	693	648	691	69	892	823	30	194
Co. Westmeath	1095	783	616	663	88	961	905	15	129
Co. Kerry	1086	661	546	673	111	856	793	10	226
Co. Waterford	1051	673	575	661	129	833	757	63	209
Co. Limerick	1026	663	545	613	75	833	778	41	191
Co. Offaly	816	540	487	533	46	705	668	13	96
Co. Clare	763	476	378	492	50	643	604	5	116
Co. Laois	736	512	461	459	55	640	616	12	89
Co. Sligo	689	449	368	489	64	604	554	18	82
Co. Kilkenny	671	420	456	458	33	580	535	24	86
Co. Cavan	626	409	343	407	37	540	492	12	83
Co. Carlow	603	422	350	396	45	527	498	17	71
Co. Roscommon	599	365	295	397	44	487	422	14	110
Co. Longford	435	293	237	305	24	374	351	4	57
Co. Monaghan	362	229	206	212	21	290	277	3	63
Co. Leitrim	350	205	167	211	29	272	257	5	74

Note: Multiple retrofits may be undertaken by each applicant if they receive the grant.

Model 2 in Table 5 shows that a larger number of (planned) retrofit measures, which is an indicator of retrofit intensity, reduces the probability of abandonment. The predicted probability of abandonment for a dwelling where only 1 retrofit measure is planned is 9 percentage points higher compared to a dwelling where 5 retrofit measures are planned (refer predicted probability estimates in Table 6). In contrast, Collins and Curtis (2017a) find that higher retrofit intensity among more affluent households is associated with a higher probability of abandonment of applications to the retrofit subsidy scheme.

The probability of abandonment also varies by type of planned retrofit measure. Planning to undertake retrofit measures like attic insulation and ventilation is associated with a higher probability of abandonment. Ventilation retrofits increase the probability of abandonment by 6 percentage points, as shown in Table 6. It should be noted that retrofit type and intensity is decided by the grant administrator, not the grant applicant, and is based on technical criteria to maximise energy efficiency. There is also evidence that seasonality plays some role in abandonment; applications made in spring and winter have a higher probability of abandonment compared to other seasons (refer model 2 in Table 3). This is similar to the findings of Collins and Curtis (2017a). To summarise, undertaking several smaller measures is associated with a lower probability of abandonment, possibly attributable to lower levels of disruption for households.

Certain upgrade types, such as ventilation^2^ or attic insulation have a higher probability of abandonment compared to a less intensive upgrade like lighting. Retrofits incorporating ventilation are more than 4 times more likely to be abandoned compared to retrofits without ventilation measures. Attic insulation retrofits are 2.1 times more likely to be abandoned compared to retrofits without attic insulation, whereas wall insulation retrofits are 0.6 times as likely to be abandoned compared to retrofits without wall insulation (as per Table 5). It is not clear why abandonment rates are higher in these instances, but they may be associated with installation disruption (e.g., emptying attic storage).

While the duration of application processing has a statistically significant association with the probability of abandonment in both models, our preferred model 2, shows that the effect has decreased across time (see Table 3). For the years 2015–2017 a higher duration of processing was associated with a higher probability of abandonment, however this effect disappears or reverses in later years. This could be attributed to efficiency improvements in the administration of the scheme with the passage of time. This is similar to the findings of Raissi and Reames (2020) and Collins and Curtis (2017a) that inefficiencies in scheme administration can lead to higher likelihood of non-participation and abandonment. Table 3 shows that having at least one employed resident reduces the probability of abandonment by 2 percentage points. This can be linked to the evidence which supports the hypothesis that poorer socio-economic status results in higher informational barriers (Abrahamse and Steg, 2011). Households with fewer residents have a higher probability of abandonment compared to larger households. This is in line with the findings by Morton et al. (2018). In Fig. 2 we plot the predicted probabilities of abandonment that are attributed to retrofit intensity differentiated by dwelling types using the estimates from Table 5. In the first panel of Fig. 1 the dwelling type of the applicants has a significant influence on the decision to abandon. Apartments show a higher probability of abandonment compared to houses. This result is consistent with Collins and Curtis (2017a), Trotta (2018), Morton et al. (2018) and (Schleich, 2019), who argue that the barriers associated with retrofits in apartment buildings are greater than in other building types. Finally, dwellings built between 1940s and 1980s are between 0.7–0.8 times as likely to abandon their grant application compared to the reference category of dwellings built between the 1990s and 2006. There is no statistical difference in probability of abandonment of applications of earlier built dwellings (i.e., pre 1940s) compared to the reference category. Again, it is not clear why dwellings in the 1940s–1980s vintage have a lower likelihood of abandonment though as illustrated in Fig. 2, the maximum difference in predicted probabilities of abandonment across construction years is relatively small at less than 2 percentage points. Previous literature also shows an unclear relationship between age of the dwelling and likelihood of retrofits (Trotta, 2018).

As previous findings suggest, there are more similarities than differences between low and higher income households when it comes to undertaking energy efficient retrofits (Wolske, 2020). Our findings indicate that certain characteristics such as dwelling type, age of dwelling, seasonality of grant application and type of retrofit suggested for poor households influence the probability of abandonment of retrofit scheme similar to the findings for higher income households (see Collins and Curtis (2017a), Morton et al. (2018), Trotta (2018) and Schleich (2019)). However, we find evidence of significant behavioural informational and behavioural barriers for poor households based on our finding that a lower retrofit intensity proposed leads to a higher probability of abandonment among poor households echoing Abrahamse and Steg (2011) and Chen et al. (2017). This can potentially be attributed to an inability to correctly understand the benefits of proposed works. The opposite of this relationship was found in previous literature among higher income households (see Collins and Curtis (2017a)).

### 3.2 Post-works efficiency gain

The pre-works energy rating was available for 2,447 observations only. The dependent variable in this case is the difference between pre- and post-works BER denominated in kWh/m^2^/year. A large positive value for change in BER indicates significant energy efficiency improvement. The results of regression analysis, estimated by ordinary least squares (OLS), are reported in Table 8. The first model estimates energy efficiency improvement as a function of total expenditure on retrofits. The second model itemises expenditure by retrofit type, which illustrates how the benefit of efficiency improvements varies by expenditure across retrofit measure types. We control for dwelling characteristics including pre-works BER rating, year of construction, type and size of the dwelling.

**Table 8:** Factors affecting energy efficiency improvements from retrofits.

	Dependent variable: change in energy rating	
	1	2
Pre-works BER (ref cat: B)		
C	11.38	18.52
	(21.60)	(21.08)
D	33.98	41.49*
	(21.73)	(21.20)
E	71.07***	79.66***
	(22.14)	(21.60)
F	114.8***	122.7***
	(22.67)	(22.12)
G	277.6***	282.3***
	(22.66)	(22.10)
Dwelling type		
Apartment	-23.87	-21.56
	(25.52)	(24.90)
Others	-4.289	-0.781
	(5.909)	(5.767)
Dwelling size	-0.179***	-0.0233
	(0.0524)	(0.0534)
Year of construction (ref cat: Before 1920s)		
1920s-1930s	22.91*	21.27*
	(12.98)	(12.66)
1940s-1960s	9.368	14.63
	(10.78)	(10.51)
1960s-1980s	10.97	15.09
	(10.10)	(9.909)
1990s-2006	18.88*	21.03**
	(10.43)	(10.27)
Log of total invoice amount	22.09***	
	(1.996)	
Log of invoice amount by category		
Heating system		12.08***
		(0.904)
Attic insulation		2.347***
		(0.802)
Lighting		-8.265
		(6.580)
Ventilation		0.719
		(1.949)
Wall insulation		1.940***
		(0.648)
Miscellaneous		-5.440***
		(1.082)
Constant	-149.5***	-3.852
	(28.59)	(33.34)

Observations	2,447	2,447
R-squared	0.489	0.515

Estimated coefficients are given in the table. Standard errors in parentheses. *** p<0.01, ** p<0.05, * p<0.1.

As anticipated higher grant expenditure is associated with higher improvement in energy efficiency rating after controlling for the initial BER. Expenditure is measured in natural logarithms, meaning that the 22.09 coefficient (model 1, Table 8) can be interpreted as meaning that on average BER improves by 0.22 kWh/m^2^/year for a 1% increase in grant expenditure. This shows that the scheme in general succeeds in improving the energy efficiency of the applicant dwellings. Properties with the lowest pre-works BER rating, labelled ‘G’, experience an improvement of 277 kWh/m^2^/year in energy efficiency relative to the reference category of those with a ‘B’ BER, controlling for other attributes. The corresponding figure for ‘C’ rated properties is just 11 kWh/m^2^/year, though not statistically different than improvements experienced by ‘B’ rated properties. The coefficient estimates associated with ‘E’, ‘F’ and ‘G’ rated properties are all statistically significant, indicating greater levels of improvement among the least energy efficient properties. This shows that the grant scheme is achieving the greatest energy efficiency improvements within the most energy inefficient properties, a finding consistent with other energy retrofit grant studies (Hoicka et al., 2014; Webber et al., 2015; Collins and Curtis, 2017b; Liddell, 2015; Elsharkawy and Rutherford, 2018).

Model 2 in Table 8 considers grant expenditures across retrofit types. The highest improvement in energy efficiency per unit grant expenditure is associated with heating system upgrades, followed by attic insulation, followed by wall insulation. This finding is in line with the findings of Collins and Curtis (2017b) for grants targeting higher income households. For heating system upgrades a 1 percent increase in grant expenditure, on average, is associated with 0.12 kWh/m^2^/year improvement in BER rating. Coefficient estimates related to lighting and ventilation retrofits are not statistically significant. In the case of ventilation, the retrofits may reflect a necessity for health and safety purposes, rather than a measure that directly improves energy efficiency. The negative and statistically significant coefficient on the ‘Miscellaneous’ retrofit measure (-5.440) is unexpected. In practice expenditure on items such as draught proofing or insulation jackets on water cylinders would not lead to a deterioration in measured energy efficiency so the negative coefficient may possibly reflect omitted variable bias.

Irrespective of retrofit measure type, it should be noted that this analysis only considers quantitative energy efficiency improvements and other benefits such as those related to comfort and health are not considered. Further research is needed to measure and understand these non-energy benefits, including reduced condensation or better overall comfort (see Kerr et al., 2017).

## 4 Conclusion and policy implications

The multidimensional benefits of improving energy efficiency in the dwellings of low-income households can be significant to the lives of the dwellers and important to the emission reduction strategies of many nations across the world. Some of the most important barriers faced by low-income households towards energy efficiency include economic and informational barriers (Sorrell et al., 2000; Crandall-Hollick and Sherlock, 2018; Bird and Hernández, 2012). Even in the absence of these impediments, low-income households face significant difficulties in getting retrofits undertaken (Raissi and Reames, 2020; Reames, 2016). The programme considered in this study is a targeted initiative to improve energy efficiency of dwellings of vulnerable households. Since the scheme offers free retrofits for qualifying dwellers, participation in the scheme by such households should be high. However, we observe that 9% of eligible households abandon their retrofit application. This rate of abandonment is lower compared to abandonment rates in partially subsidised energy retrofit schemes such as Better Energy Homes Scheme by SEAI in Ireland with a 15% rate of abandonment (Collins and Curtis, 2017a). Making an application implies that the informational barriers are low and that occupants are motivated to have their homes retrofitted. The grant scheme itself obviates any financial barriers. Hence, we investigate the interrelationship between application abandonment and economic and dwelling related characteristics available within the administrative dataset associated with the grant scheme. We add to a sparse literature on energy retrofits in low-income or social housing, which is characterised by very small sample sizes (see for example Beagon et al., 2018; Hernández and Phillips, 2015). We find that the total number of (planned) retrofits measures play an important role in determining the probability of abandonment. A higher retrofit intensity could be perceived as higher energy efficiency improvement by the applicants and hence this leads to a higher probability of them proceeding with the retrofits. A lower number of planned retrofit measures is associated with a higher probability of abandonment, which might be attributed to households perceiving fewer retrofit measures as having lower potential benefits. A higher measure intensity may not necessarily translate to a bigger improvement in energy efficiency since some of the retrofit activities undertaken are ancillary to the main retrofit. This finding is consistent with the literature on behavioural and informational barriers that there may be an inability to correctly assess the long term monetary and environmental benefits of retrofits (Chen et al., 2017; Day and Gunderson, 2015). Focusing on expanding the consulting and energy advice components of the schemes, particularly in the early stages, to clearly convey the benefits of energy efficiency retrofits to the occupants may help reduce abandonment rates, as found elsewhere (Achtnicht and Madlener, 2014). Ramsden (2020) shows that advice related to energy efficiency is helpful to vulnerable households when the advice is provided in coordination between government and charities. Seasonality also plays a role in the abandonment of applications. Winter and spring applications have higher levels of abandonment compared to other times, which confirms findings in previous studies that disruption due to retrofits, especially in colder months, can be a deterrent to successful completion of retrofits (Collins and Curtis, 2017a). Simple practical measures during retrofit planning stage, such as the deferral of retrofit works, may be able to minimise abandonment associated with the season.

While it is important to assess the effectiveness of schemes like BEWHS in improving energy efficiency of low-income households, assessing the gains from the scheme as a whole requires comprehensive data on all multi-dimensional benefits the occupants may derive from the retrofits. In this study we focus on the effectiveness of the scheme in terms of energy efficiency improvements achieved by a subset of applicant households. The analysis shows that grant expenditure on retrofits delivers energy efficiency improvements, but the magnitude of improvement varies depending on initial building energy efficiency, as well as retrofit measure types. The greatest value for money from grant expenditure occurs among dwellings with the poorest pre-works energy efficiency. Additionally, retrofits such as heating system upgrades also yield among the highest energy efficiency improvements. However, planned retrofits that comprise ventilation works are four times more likely to be abandoned compared to those without ventilation works. Ventilation retrofits are advised for health and safety reasons, but such a high associated level of abandonment indicates that greater effort is necessary to convey to occupants the necessity and benefits of improved ventilation within homes. Aside from finance, a lack of comprehension in this area may represent the most significant barrier to households undertaking energy efficiency retrofits. Otherwise, the analysis shows that from an energy efficiency perspective, the scheme is making a significant difference in the quality of dwellings of low-income households. Other benefits of the scheme, such as improve comfort and health outcomes, as well as lower energy costs are also likely to arise (Hernández and Phillips, 2015).

While our research utilises an administrative dataset with a larger sample size compared to similar studies, we cannot control for environmental awareness of applicants or their attitudes towards energy costs in this study due to lack of data. Future studies can bring a more holistic analysis if such data is collected by administering agencies in any pre-retrofit stages. A spatial approach will be more suitable to understand the social network effects of grant application and completion of retrofits by low-income households. While evaluating the benefits of the grant, it is important to consider the yearly energy savings and health impacts arising from the improvements in home condition.

## Declarations

### Author contribution statement

Arya Pillai, Miguel Reanos & John Curtis: Conceived and designed the experiments; Performed the experiments; Analyzed and interpreted the data; Contributed reagents, materials, analysis tools or data; Wrote the paper.

### Funding statement

This work was supported by the 10.13039/501100001603Sustainable Energy Authority of Ireland (19/RDD/427) and by the 10.13039/501100001602Science Foundation Ireland (MaREI - 12/RC/2303).

### Data availability statement

The authors do not have permission to share data.

### Declaration of interests statement

The authors declare no conflict of interest.

### Additional information

No additional information is available for this paper.

## Footnotes

1 For instance, the type of retrofit activities prescribed by SEAI after 2018 has changed to include deeper retrofits activities (retrofits which bring a larger energy efficiency improvement) compared to the pre-2018 scheme administration.

2 Ventilation related retrofit includes roof ventilation, wall vents, magnetic filtration device, mechanical extract vent, air tightness testing etc.
